# Ultrasound-Triggered Effects of the Microbubbles Coupled to GDNF Plasmid-Loaded PEGylated Liposomes in a Rat Model of Parkinson's Disease

**DOI:** 10.3389/fnins.2018.00222

**Published:** 2018-04-06

**Authors:** Peijian Yue, Wang Miao, Lin Gao, Xinyu Zhao, Junfang Teng

**Affiliations:** ^1^Department of Neurology, The First Affiliated Hospital of Zhengzhou University, Zhengzhou, China; ^2^Department of Neurological Intensive Care Unit, The First Affiliated Hospital of Zhengzhou University, Zhengzhou, China

**Keywords:** GDNF, microbubbles, Parkinson's disease, PEGylated liposomes, ultrasound

## Abstract

**Background:** The purpose of this study was to investigate ultrasound-triggered effects of PEGylated liposomes-coupled microbubbles mediated gene transfer of glial cell line-derived neurotrophic factor (GDNF) plasmid (PLs-GDNF-MBs) on behavioral deficits and neuron loss in a rat model of Parkinson's disease (PD).

**Methods:** The unloaded PLs-MBs were characterized for particle size, concentration and zeta potential. PD rat model was established by a unilateral 6-hydroxydopamine (6-OHDA) lesion. Rotational, climbing pole, and suspension tests were used to evaluate behavioral deficits. The immunohistochemical staining of tyrosine hydroxylase (TH) and dopamine transporter (DAT) was used to assess the neuron loss. The expression levels of GDNF and nuclear receptor-related factor 1 (Nurr1) were determined by western blot and qRT-PCR analysis.

**Results:** The particle size of PLs-MBs was gradually increased, while the concentration and absolute zeta potential were gradually decreased in a time-dependent manner after injection. 6-OHDA elevated amphetamine-induced rotations and decreased the TH and DAT immunoreactivity compared to sham group. However, these effects were blocked by the PLs-GDNF-MBs. In addition, the mRNA and protein expression levels of GDNF and Nurr1 were increased after PLs-GDNF-MBs treatment.

**Conclusions:** The delivery of PLs-GDNF-MBs into the brains using MRI-guided focused ultrasound alleviates the behavioral deficits and neuron loss in the rat model of PD.

## Introduction

Parkinson's disease (PD) is a common neurodegenerative disease, which is characterized by the loss of dopaminergic (DA) neurons in the substantia nigra (SN) (Proft et al., 2011). Patients with PD have some typical symptoms, including tremor, rigidity, and bradykinesia (Kim et al., 2011). Although multiple attempts have been made to establish therapies for PD, neither a cure nor a treatment has been proven to slow progression (AlDakheel et al., 2014). Therefore, more efforts are needed to expand the therapeutic strategies.

Glial cell line-derived neurotrophic factor (GDNF) is considered as an essential neuroprotective ligand for midbrain DA neurons (Nitta et al., 2004). It is expressed throughout the central nervous system (CNS) during development (Nosrat et al., 1996). GDNF promotes the survival, function, and neurite growth of DA neurons both *in vitro* and *in vivo* (Kumar et al., 2015). Many studies on animal models of PD have reported beneficial effects of GDNF on DA neuron survival (Patel et al., 2013; Quintino et al., 2013). Since GDNF does not cross the blood-brain barrier (BBB), intracerebral gene delivery by injection of viral vectors has been considered as a method of administration (Tenenbaum and Humbert-Claude, 2017). However, intracerebroventricularly administered GDNF showed numerous negative side effects and no prominent clinical improvements (Nutt et al., 2003). New delivery methods for GDNF are being investigated.

Magnetic resonance imaging (MRI)-guided focused ultrasound (FUS) is a noninvasive method to induce transient BBB disruption by increasing the temperature or creating gas bubbles in the targeted tissues (Huang Q. et al., 2012). Microbubbles (MBs) consist of a gas core stabilized by a thin shell (Deelman et al., 2010). MBs are becoming increasingly popular tools for targeted drug delivery (Sheffield et al., 2008). Ultrasound-mediated microbubble destruction technology has become a relatively safe and promising method of gene delivery in recent years (Phillips et al., 2010). Previous studies have revealed that FUS combined with microbubbles were able to induce a targeted and reversible BBB opening in rats (Deng et al., 2012), mouse (Zhao et al., 2018), rabbits (Hynynen et al., 2001), and pigs (Huang et al., 2017). Until now, various neurotrophic factors (Samiotaki et al., 2015) and genes (Lin et al., 2016) have been delivered into targeted brain regions by FUS mediated MBs to treat neurodegenerative disease. However, the DNA-carrying capacity of MBs is limited (Zhou et al., 2015).

Polyethylene glycol (PEG)ylated liposomes (PLs) are promising and valuable drug carriers due to their many advantages including good biocompatibility, good stability, and high DNA-carrying capacity (Huang, 2008; Chen et al., 2013). However, the steric barrier of the grafted PEG moiety on the surface of the liposomes reduces interaction between the delivery system and the cell surface (Chen et al., 2011). Thus, coupling gene-loaded liposomes to the microbubble surface is adopted (Lentacker et al., 2010). This approach takes full advantage of the high DNA-carrying capacity of PLs and the ultrasound-mediated BBB disruption effect of MBs (Wang et al., 2014).

The purpose of this study was to explore ultrasound-triggered effects of the GDNF plasmid gene loaded-PEGylated liposomes-coupled microbubbles (PLs-GDNF-MBs) on behavioral deficits and neuron loss in a rat model of PD.

## Materials and methods

### Preparation of GDNF-loaded pegylated liposomes

PEGylated liposomes (Avanti Polar Lipids Inc., USA) containing appropriate amounts of soybean phosphatidylcholine (S100-PC), 3beta [N-(N′, N′-dimethylaminoethane)-carbamoyl] cholesterol (DC-cholesterol) and biotinylated 1,2-distearoyl-sn-glycero-3-phosphoethanolamine-n-(carboxy[polyethylene glycol]-2,000) (Bio-PEG2000-DSPE) at a molar ratio of 90:10:5 were prepared using a thin-film hydration method (Meure et al., 2008). In brief, the lipids were dissolved in chloroform and kept overnight on a rotary evaporator (RE-52C; Shanghai Yaguang Instrument Co Ltd, Shanghai, China) at 40°C for solvent evaporation. After evaporation, a thin lipid film was formed and dried further in a vacuum for 8 h to remove the residual solvent. The lipid film was hydrated for 8 h using 2.4 ml sterile phosphate-buffered solution (PBS) containing pDC315-GDNF plasmid (5 μg) which was constructed by inserting GDNF cDNA of rat into pDC315 vector (Microbix Biosystems Inc., Toronto, Canada). The dispersion was sonicated for 5 min and then extruded 12 times through a polycarbonate membrane (100 nm pore size) using a mini-extruder (Avanti Polar Lipids Inc., USA). The plasmid was separated from the encapsulated one by Sepharose CL-4B column chromatography.

### Preparation of microbubbles

Microbubbles were prepared according to the procedures described previously (Liu et al., 2011). Briefly, 1,2-distearoyl-sn-glycero-3-phosphocholine (DSPC) (5 mg), DSPE-PEG2000 (0.5 mg), Bio-PEG2000-DSPE (1 mg), glycerol (50 μl), and PBS (450 μl) were mixed in a glass container. The mixture was placed in a 60°C water bath for 30 min. The lipid suspension was subsequently lyophilized. The dried powder was dispersed in 1 ml of a liquid mixture of 50% glucose, propylene glycol, and glycerin in a volume ratio of 8:1:1. Then the mixture was sonicated for 30 s in the presence of perfluoropropane (C_3_F_8_) gas. Subsequently, it was mechanically vibrated at 4 kHz for 50 s in a dental amalgamator (YJT Medical Apparatuses and Instruments, Shanghai, China). The generated biotinylated microbubbles were washed and resuspended in PBS. Microbubbles were avidinylated by incubating with avidin (0.025 mg of avidin per ml of microbubbles; Cell Sciences, Canton, USA) for 5 min.

### Coupling of biotinylated liposomes to avidinylated microbubbles

The biotinylated liposomes (one vial) were mixed with avidinylated microbubbles (2 ml) and incubated at room temperature for 10 min. The liposome-coupled microbubbles were washed with PBS and stored at −20°C for further use.

### Characterization of PEGylated liposomes-microbubbles (PLs-MBs) complexes

The particle size and zeta potential of PLs-MBs complexes were determined according to previous reports (Matos et al., 2004). The PLs-MBs complexes (100 μl) were diluted with deionized water (900 μl) and were measured by the dynamic light scattering using a zeta potential/particle sizer Nicomp 380 ZLS (Particle Sizing Systems, Santa Barbara, CA).

The concentration of PLs-MBs complexes was measured by ultraviolet absorbance (Dewitte et al., 2015). Briefly, 2 ml of the PLs-MBs complexes was dissolved in 8 ml ethanol. After vortexing for 10 min, this suspension was filtered with polycarbonate membrane filters with 0.22 μm pores to remove undissolved materials. The ultraviolet absorbance of each sample was then measured at 458 nm (UV-2401PC, Shimadzu, Japan).

### Animals

Forty healthy Sprague-Dawley rats (10 rats per group; 200–250 g), half male and half female, were used in this experiment. Rats were housed in a temperature- and humidity controlled environment under specific pathogen-free conditions. Rats were given access to water and food *ad libitum*. The experimental design was approved by the Ethics Committee of The First Affiliated Hospital of Zhengzhou University. All animal experimental procedures were approved according to the guidelines of the Care and Use of Laboratory Animals by the National Institute of Health, China.

### 6-OHDA lesion surgery

The rats received a unilateral 6-OHDA lesion of the right substantia nigra (SN) (Metz and Whishaw, 2002). The rats were anesthetized by intraperitoneal injection of 10% chloral hydrate. After that, the rats were placed in a stereotaxic apparatus (Stoelting Instruments, Wood Dale, IL; USA). Then 6-OHDA [5 μg/per rat in 2 μl saline with 0.2% (w/v) ascorbic acid] were injected into the SN of rats at the following stereotaxic coordinates: 5.2 mm posterior to bregma, 1.0 mm lateral to the midline, and 8.5 mm ventral to the skull surface. Then 6-OHDA was infused by infusion pump at the flow rate of 1 μl/min. Sham-operated rats received the same procedure except 2 μl vehicle of 6-OHDA (0.9% saline containing 0.2% ascorbic acid) was infused into the SN. After surgery, the skin was sutured and cleaned, and the rats were allowed to recover on a heating pad before they were returned to their home cage. After 1 week, the PLs-MBs were given to rats.

### MRI-guided focused ultrasound (FUS)

Thirty rats with PD prepared as described above were randomly divided into three groups (*n* = 10) as follows: PBS, PLs-MBs+FUS, and PLs-GDNF-MBs+FUS. Apart from the PBS group, the rats were all undergone MRI-guided-FUS.

Animals were anesthetized and placed supine on a positioning sled for MRI. Brain ultrasound treatment was conducted under the following settings: 1 MHz, 20% duty cycle, and an ultrasound intensity of 2 W/cm^2^ (Wang et al., 2014). After injecting PLs-MBs (0.01 ml/kg body weight) and PLs-GDNF-MBs (0.01 ml/kg body weight) into the tail vein for 30 min, FUS was performed. The brain of each rat was sonicated from the dorsal surface into the right hemisphere at a depth of 2-3 mm. After the sonication procedure, Evans blue (EB) (100 mg/kg; Sigma, USA) was injected through the tail vein to confirm the site of BBB disruption on tissue blocks.

Animals were intravenously injection of the PBS, PLs-MBs, and PLs-GDNF-MBs once every 3 days. The rats were sacrificed at 3 weeks after treatment.

### Behavior tests

#### Rotational behavior test

Rotational behavior was performed as previously described (Pranski et al., 2013). Animals received 2.5 mg/kg D-amphetamine intraperitoneally. One hour after injection, rats were monitored for rotation behavior for 30 min. Rotations ipsilateral to the lesion were counted as a positive value, while rotations toward the contralateral side of the lesion were counted as a negative value.

#### Suspension experiment

The test was performed as previously reported (Meng et al., 2017). Briefly, the rats were placed on a horizontal wire of ~1.5 mm in diameter, suspended 30 cm from the ground. The hang time was recorded to detect rat limb coordination. Scoring criteria: 0–5 s, 0 points; 6–10 s 2 points; 11–15 sec, 3 points; 16–20 s, 4 points; and >20 s, 5 points. The mean values were calculated.

Climbing pole test: The climbing pole test was conducted as previously described (Liu et al., 2016). Briefly, a rat was placed at the top of a pole with a length of 60 and 1.0 cm in diameter. The times for climbing down the upper half of the pole, for climbing down the lower half of the pole and for climbing down the total length of the pole were recorded. And the results were scored as 3, within 3 s; 2, within 6 s and 1, over 6 s. The results were expressed as the total score.

### Immunohistochemistry

Animals were sacrificed via pentobarbital overdose (60 mg/kg) and intracardially perfused with saline, then 4% paraformaldehyde. Brains were removed and post-fixed in 4% paraformaldehyde for 24 h and then dehydrated in 30% sucrose. The sections at a thickness of 16 μm were cut through the substantia nigra (−2.92 to −3.88 mm from bregma) (Paxinos and Watson, 2007). The tissue sections were incubated in 0.3% H_2_O_2_ for 45 min, and blocked in 10% goat serum for 1 h. The sections were incubated overnight at 4°C with rabbit anti-tyrosine hydroxylase (TH; 1:300; Millipore), rabbit anti-dopamine transporter (DAT; 1:200; Santa Cruz), rabbit anti-GDNF (1:200; Santa Cruz Biotechnology) or rabbit anti-Nurr1 (1:100; Santa Cruz Biotechnology). Subsequently, the sections were incubated with biotinylated goat anti-rabbit IgG antibody (1:250; Vector labs) and avidin-biotin complex (ABC-kit, Vector Labs) at 37°C for 30 min, and then stained with 3,3′-diaminobenzidine (DAB). Then the sections were observed under a light microscope (BX51; Olympus, Tokyo, Japan). Image scanning analysis system (Image-Pro Plus) was used to analyze the changes in integrated optical density (IOD) of DAT, TH and GFAP.

### Quantitative real-time PCR (qRT-PCR)

Total RNA was isolated using Trizol (Invitrogen, Carlsbad, CA) from the frozen brains of rats. cDNA synthesis was performed using a High Capacity cDNA Reverse Transcription Kit (Applied Biosystems, USA) according to the manufacturer's instructions. QRT-PCR was performed by using SYBR green master PCR mix (Applied Biosystems, USA) on a 7,500 real-time PCR system (Applied Biosystems). The glyceraldehyde-3-phosphate (GAPDH) was used as an internal control. Primers used for analyses were listed below.

Gdnf-F: 5′-CGGACGGGACTCTAAGATGA-3′

Gdnf-R: 5′-CGTCATCAAACTGGTCAGGA-3′

Nurr1-F: 5′-CAACTACAGCACAGGCTACGA-3′

Nurr1-R: 5′-GCATCTGAATGTCTTCTACCTTAATG-3′

Gapdh-F: 5′-GTGAAGGTCGGTGTCAACGGATTT-3′

Gapdh-R: 5′-CACAGTCTTCTGAGTGGCAGTGAT-3′

### Western blot analysis

Tissue samples were lysed in RIPA buffer in the presence of protease inhibitors (Roche, USA). Equal amounts of proteins (50 μg) were separated on 10% SDS-polyacrilamide gel and then transferred to PVDF membranes (Amersham). After being blocked in 5% non-fat milk for 1 h, the membrane was incubated overnight at 4°C with the following primary antibodies: anti-GDNF (1:1,000; R&D Systems), anti-Nurr1 (1:1000; Santa Cruz, USA), and anti-GAPDH (1:500; Santa Cruz, USA). After washing with TBST, the membranes were incubated with peroxidase-conjugated secondary antibody (1:10,000, Cell Signaling Technologies) for 1 h at 37°C. The immunoreaction was visualized with enhanced chemiluminescence plus reagents (Millipore, USA). GAPDH was used as an internal control.

### Statistical analysis

All data are presented as means ± SD. All statistical analyses were performed using GraphPad Prism 6.0 software (GraphPad Prism Inc., USA). Comparison between two groups was performed by unpaired Student's *t*-test. Comparison among multiple groups was performed by one-way analysis of variance (ANOVA) with LSD *post hoc* test. *P* < 0.05 was considered statistically significant.

## Results

### The properties of unloaded PLs-MBs

**Figure 1:**
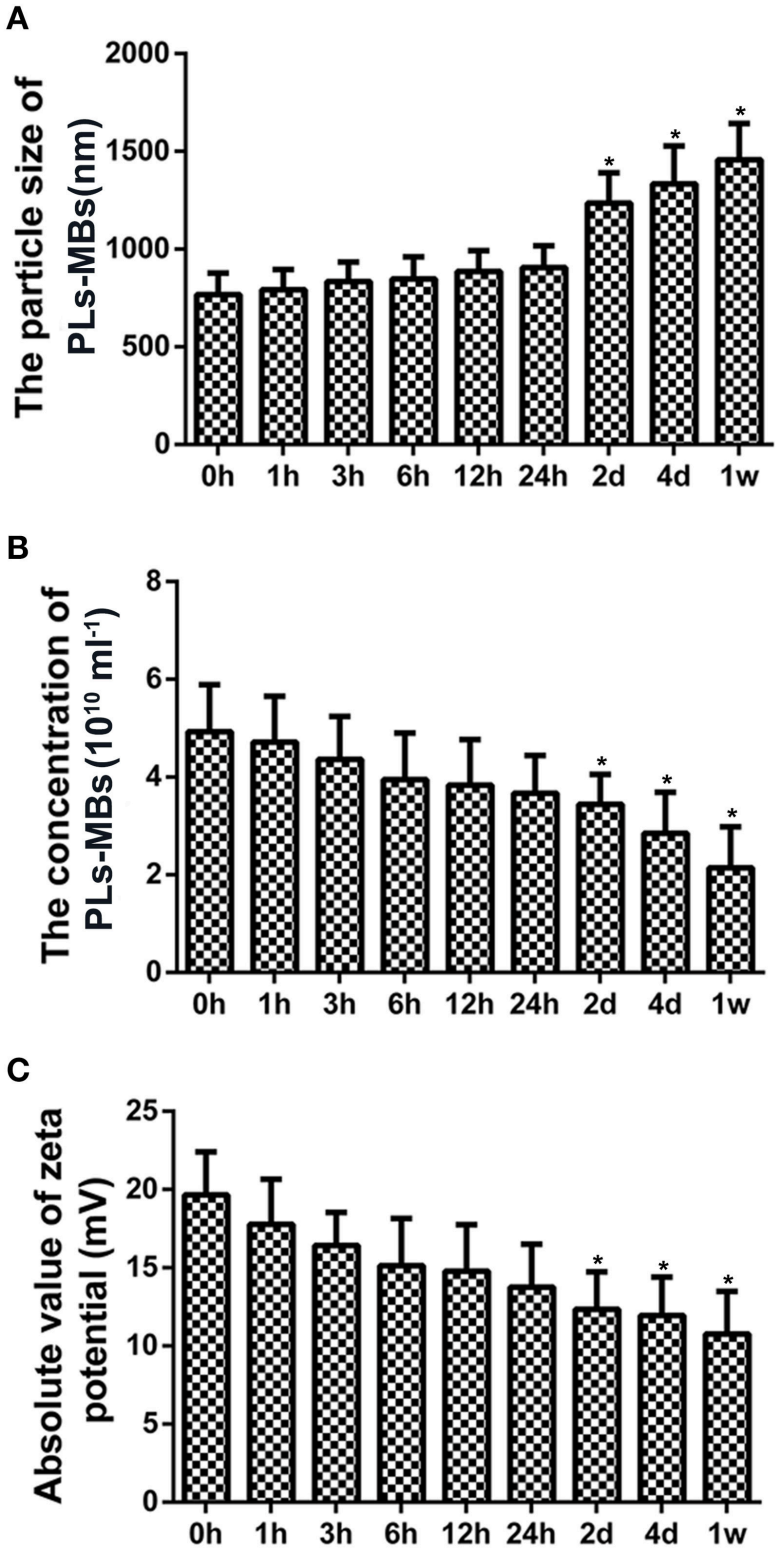
Characterization of unloaded PLs-MBs. **(A)** Size, **(B)** Concentration, and **(C)** zeta potential of unloaded PLs-MBs for different duration. *P < 0.05 vs. 0 h group.

Particle size, concentration, and zeta potential were measured for analysis of the properties of unloaded PLs-MBs. At 0 h, the mean particle size of PLs-MBs was 768.24 ± 108 nm with a mean concentration of 4.93 ± 0.96 × 10^10^/ml, and its mean absolute value of zeta potential was 19.66 mV ± 2.75 mA (Figures 1A–C). With the extension of time, the particle size was gradually increased, while the concentration and absolute zeta potential were gradually decreased, suggesting the stability of PLs-MBs is gradually decreased as the time prolongs. The differences in the particle size, concentration and zeta potential became significant at day 2, the mean particle size of PLs-MBs was 1235.62 ± 154 nm with a mean concentration of 3.44 ± 0.62 × 10^10^/ml, and its mean absolute value of zeta potential was 12.35 mV ± 2.38 mA (Figures 1A–C). The ever-increasing size and decreasing zeta potential could inhibit the effect of PLs-MBs, which needs to be improved.

### The behavioral deficits and neuron loss in a rat model of PD

**Figure 2:**
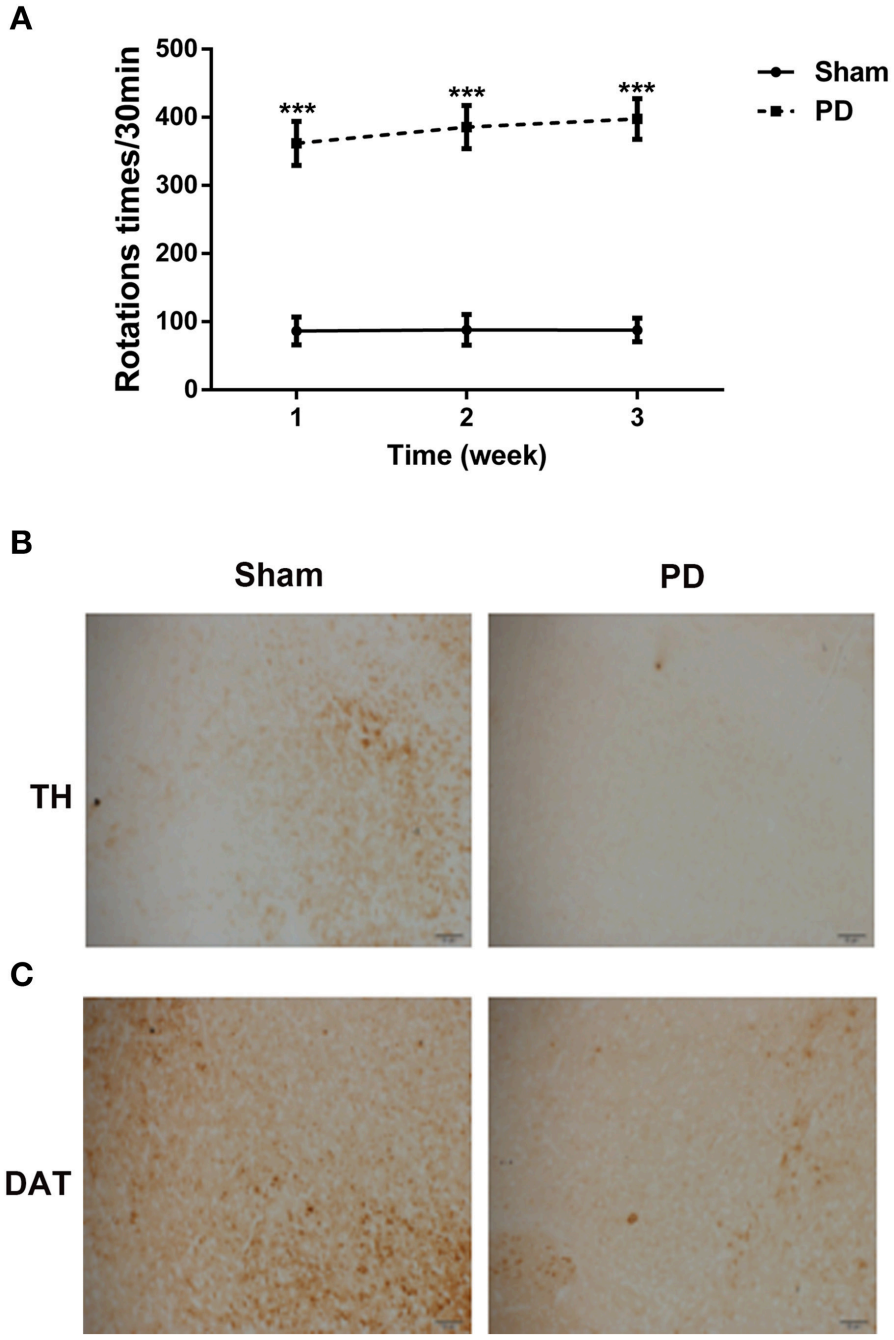
The behavioral deficits and neuron loss in rats after unilateral injections of 6-OHDA into their SN. **(A)** The asymmetric rotational behavior induced by amphetamine after 6-OHDA injection for 1, 2, and 3 weeks. **(B)** Representative immunostainings of TH protein in the SN after 6-OHDA injection for 3 weeks. Scale bars = 50 μm. **(C)** Representative immunostainings of DAT protein in the SN after 6-OHDA injection for 3 weeks. Scale bars = 50 μm. ***P < 0.001 vs. Sham group.

To assess the effects of 6-OHDA treatment on behavioral deficits, rotational behavior test was performed. As shown in Figure 2A, the number of rotations gradually increased in the PD group in a time-dependent manner when compared to sham group.

To clarify the effect of 6-OHDA on DA neuron loss, immunohistochemical staining for TH and DAT proteins were performed. Three weeks after surgery, 6-OHDA resulted in an obvious decrease in the TH and DAT protein expression levels in comparison with sham group (Figures 2B,C), indicating that the PD rat model was established successfully.

### PLs-GDNF-MBs alleviated the behavioral deficits and neuron loss

**Figure 3:**
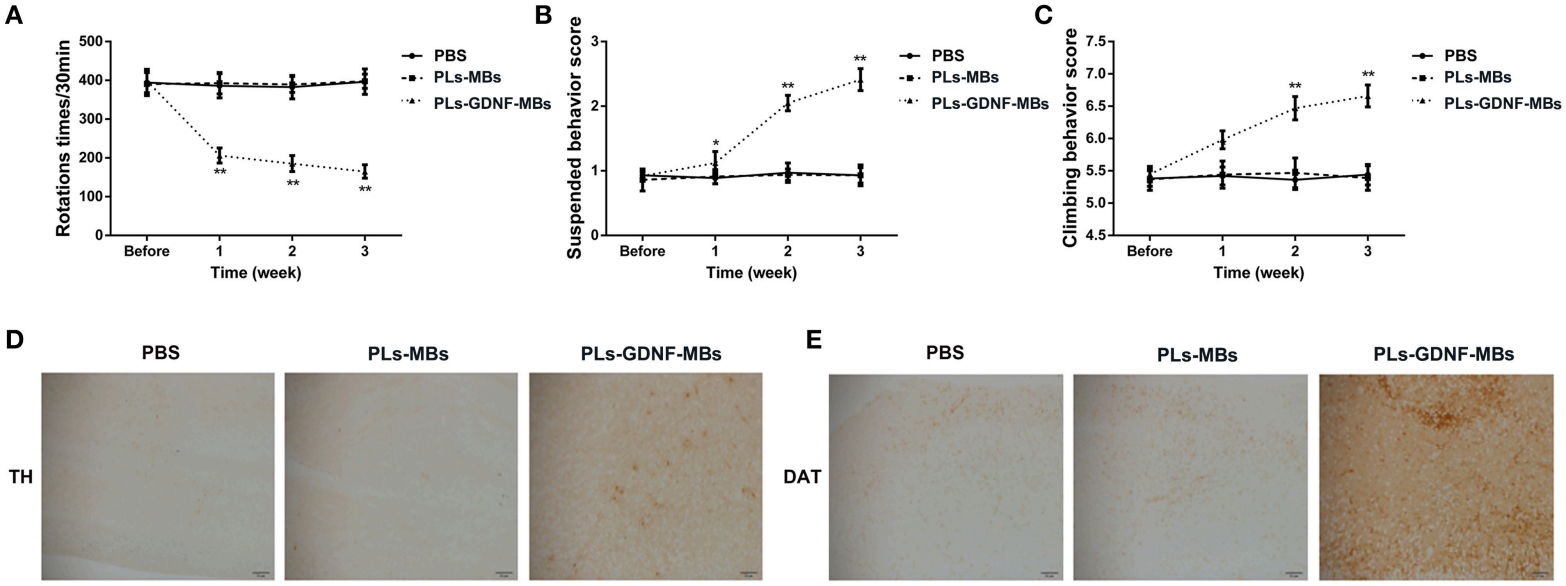
Effects of PLs-GDNF-MBs on the behavioral changes and neuron loss in PD rats. **(A)** The asymmetric rotational behavior induced by amphetamine before and after PLs-GDNF-MBs injection for 1, 2, and 3 weeks. **(B)** Scores of suspension test and **(C)** climbing pole test before and after PLs-GDNF-MBs injection for 1, 2, and 3 weeks. **(D)** Representative immunostainings of TH protein in the SN of PD rats after PLs-GDNF-MBs injection for 3 weeks. Scale bars = 50 μm. **(E)** Representative immunostainings of DAT protein in the SN of PD rats after PLs-GDNF-MBs injection for 3 weeks. Scale bars = 50 μm. *P < 0.05 vs. PLs-MBs group, **P < 0.01 vs. PLs-MBs group.

To evaluate the effects of PLs-GDNF-MBs on 6-OHDA-induced behavioral deficits, PLs-GDNF-MBs were injected intravenously and delivered to the 6-OHDA lesioned striatum with MRI-guided FUS one week after unilateral partial lesioning with 6-OHDA and then the rats received three behavioral tests at 1, 2, and 3 weeks following PLs-GDNF-MBs injection. In the three behavioral experiments, there were no significant differences between PBS and PLs-MBs groups (Figures 3A–C). In Figure 3A, the apomorphine-induced rotational behavior was significantly reduced after PLs-GDNF-MBs treatment as compared to the PLs-MBs group. Besides, PLs-GDNF-MBs significantly up-regulated the scores in suspension and climbing pole tests when compared to PLs-MBs group (Figures 3B,C).

Subsequently, we detected the effects of PLs-GDNF-MBs on 6-OHDA-induced neuron loss. Treatment with PLs-MBs had no effect on TH and DAT immunoreactivity in the SN compared with PBS group (Figures 3D,E). By contrast, higher TH and DAT immunostaining in the SN of the rats injected with PLs-GDNF-MBs compared to the animals treated with PLs-MBs, suggesting the PLs-GDNF-MBs attenuated the 6-OHDA-induced neurotoxicity.

### PLs-GDNF-MBs increased the expression of GDNF and Nurr1

**Figure 4:**
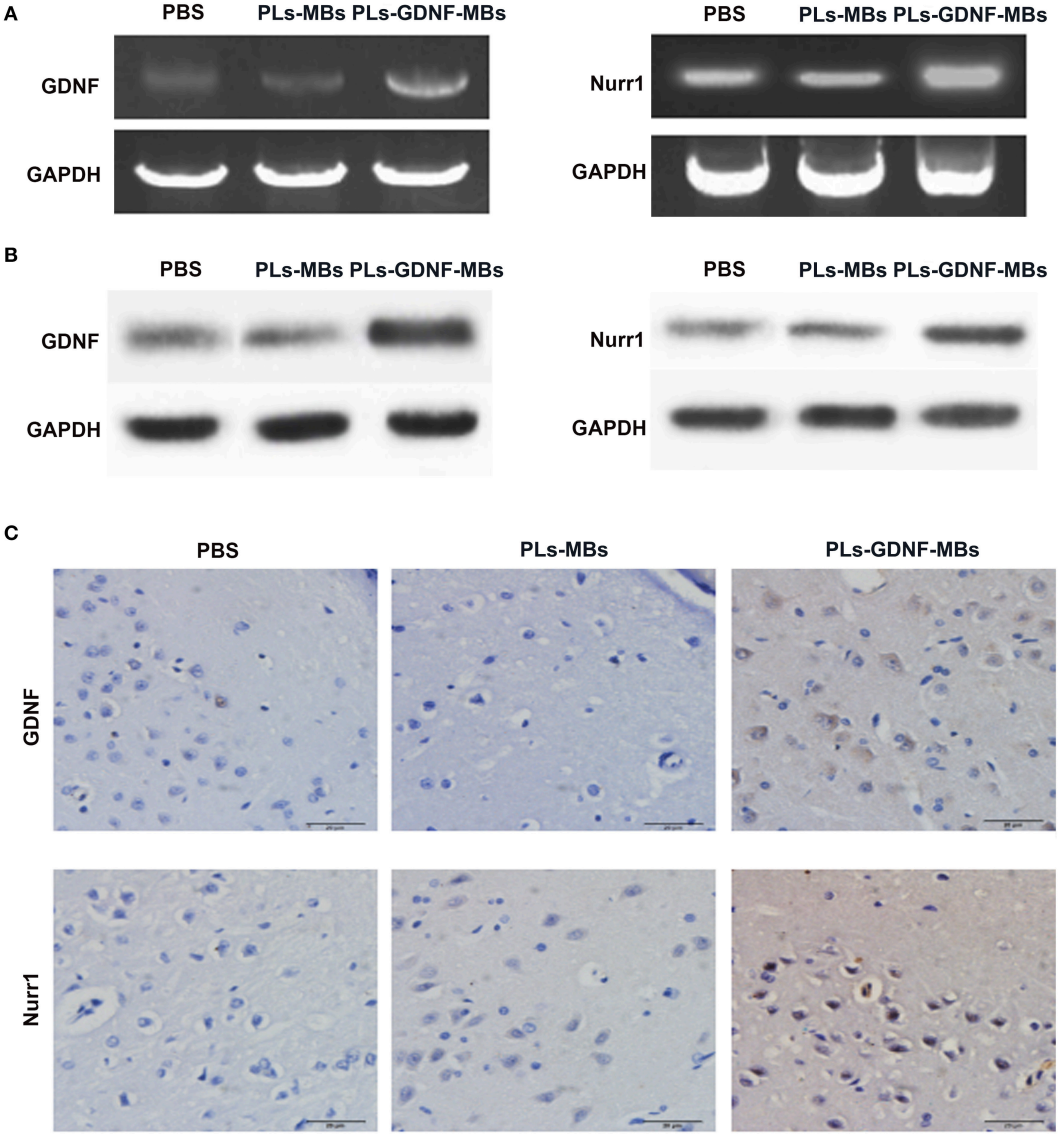
Effects of PLs-GDNF-MBs on the mRNA and protein expression levels of GDNF and Nurr1 in PD rats. **(A)** QPCR analysis of GDNF and Nurr1 mRNA levels in the brain tissues of PD rats after PLs-GDNF-MBs injection for 3 weeks. **(B)** Western blot analysis of GDNF and Nurr1 protein levels in the brain tissues of PD rats after PLs-GDNF-MBs injection for 3 weeks. **(C)** Representative immunostainings of GDNF and Nurr1 proteins in the SN of PD rats after PLs-GDNF-MBs injection for 3 weeks. Scale bars = 20 μm.

Next, we investigated the effects of PLs-GDNF-MBs on the expression of GDNF and Nurr1 *in vivo*. The results showed that the mRNA and protein expression levels of GDNF were obviously increased after PLs-GDNF-MBs treatment. In addition, the Nurr1 mRNA and protein levels also increased after PLs-GDNF-MBs treatment *in vivo* (Figures 4A,B). Furthermore, higher GDNF and Nurr1 immunostaining was observed in the PLs-GDNF-MBs group compared to the PLs-MBs group (Figure 4C). Taken together, these results suggest that Ultrasound-triggered PLs-GDNF-MBs promote both GDNF and Nurr1 expression in the protection against DA neuron loss.

## Discussion

Clinical trials of neurotrophic factor therapy for the treatment of Parkinson's disease through direct delivery of recombinant GDNF have failed to display clinical benefit (Lang et al., 2006). Moreover, great efforts have been made for the development of *in vivo* gene transfer by vectors expressing the GDNF gene (Kirik et al., 2000, 2004). However, the therapeutic application of the GDNF protein or gene in the CNS is related to a major problem: the CNS is protected by the BBB, so it is easily blocked (Wang et al., 2012).

Biochemical modifications of the nanocarriers and BBB disruption with FUS and MBs are promising approaches which enhance transport or bypass the BBB (Papademetriou and Porter, 2015). Recently, FUS combined with a specially designed plasmid-conjugated MBs have already been demonstrated the possibility of local CNS gene expression (Huang Q. et al., 2012). Hsu et al. demonstrated that the transduction of astrocytes by AAV2-GFP delivered can successfully penetrate the BBB-opened brain regions to express GFP through FUS-BBB opening (Hsu et al., 2013). Weber-Adrian et al. found that a viral vector (scAAV9-GFP) was injected intravenously and MRI-guided FUS was used to target one side of the cervical spinal cord in adult rats (Weber-Adrian et al., 2015). In our studies, as the DNA-carrying capacity of MBs is limited (Zhou et al., 2015), the PLs-MBs complexes combined with MRI-guided FUS for gene delivery were established.

It is known that reducing the particle size of delivery systems may enhance the cellular uptake of delivery systems (Kumari and Yadav, 2011). Besides, the stability of nanoparticle is very important for their practical applications, and it is closely related to its' electrokinetic properties (Huang Y. et al., 2012). Generally, nanoparticles with high absolute zeta potential are electrically stabilized and have good stability (Xu et al., 2012). Our results showed that the particle size of the PLs-MBs complexes was gradually increased, while the concentration and absolute zeta potential were gradually decreased in a time-dependent manner, suggesting the stability of PLs-MBs complexes is gradually decreased as the time prolongs. This is the deficiency of the PLs-MBs complexes, which needs further improvement.

Next, we assessed the effects of the PLs-GDNF-MBs on behavioral deficits. The apomorphine-induced rotational test was used to examine the injury degree of dopaminergic system (Tai et al., 2013). Our data showed that the number of apomorphine-induced rotations significantly increased in the PD group compared with the sham group. In contrast, PLs-GDNF-MBs treatment showed an obvious reduction in this behavioral deficit. The other two behavioral deficits were also significantly alleviated by PLs-GDNF-MBs treatment measured by climbing pole test and suspension test. These results suggest the protective effects of PLs-GDNF-MBs on attenuating behavioral deficits in the PD rat model. GDNF supports the growth and survival of dopaminergic neurons and is a potent agent for PD therapy due to its neuroprotective and neurotrophic effects (Lin et al., 1993). Previous studies have shown that GDNF plasmid gene delivery is feasible when using recombinant AAV vectors as a gene vector (Kells et al., 2012; Redmond et al., 2013).

TH, the rate-limiting enzyme in DA synthesis, is a well-established marker for identification of DA neurons (Apuschkin et al., 2015). DAT is essential for maintaining DA homeostasis and is a marker for the evaluation of the integrity of the DA system (Afonso-Oramas et al., 2010). In our study, we combined the expression of DA markers (TH and DAT) to evaluate the effects of PLs-GDNF-MBs on neuron loss. Our immunohistochemical analysis showed that TH and DAT immunostaining were lower in the PD group as compared with the sham group. However, TH and DAT immunoreactivity were higher in the animals receiving PLs-GDNF-MBs for 3 weeks, indicating the neuroprotective effects of PLs-GDNF-MBs in the rats with PD.

It has been demonstrated that GDNF promotes the expression of Nurr1 in midbrain-derived neural stem cells (Lei et al., 2011). Moreover, preclinical studies have demonstrated the essential role of Nurr1 in the midbrain DA neurons development and functional maintenance (Kadkhodaei et al., 2013). Our results showed that an increase in GDNF and Nurr1 expression levels in the PLs-GDNF-MBs group compared to the PLs-MBs group. Therefore, PLs-GDNF-MBs might relieve the neuron loss by increasing the expression of GDNF and Nurr1.

There are some limitations of this study: (1) One control appears to be missing though: what would be the effect of the infusion of GDNF-loaded microbubbles without ultrasound treatment? (2) Immunogenicity and toxicity studies should be performed because of PLs-MBs are immunogenic. (3) 6-OHDA-induced PD rats did not recapitulate all the pathologic abnormalities in PD clinical cases; (4) only one-time FUS exposure was used. Repeated FUS treatment might further enhance the effect of PLs-GDNF-MBs.

In conclusion, PLs-GDNF-MBs combined with MRI-guided focused ultrasound may be an effective way of delivering the GDNF gene directly into the brain. The method provides a potential strategy of treating patients with PD.

## Author contributions

PY and WM: designed the project; LG: performed the experiments; XZ: analyzed the data; JT: drafted the manuscript.

### Conflict of interest statement

The authors declare that the research was conducted in the absence of any commercial or financial relationships that could be construed as a potential conflict of interest.
